# Application of UHF Sensors in Power System Equipment for Partial Discharge Detection: A Review

**DOI:** 10.3390/s19051029

**Published:** 2019-02-28

**Authors:** Hua Chai, B.T. Phung, Steve Mitchell

**Affiliations:** 1 School of Electrical Engineering and Telecommunications, University of New South Wales, Sydney 2052, NSW, Australia; hua.chai@unsw.edu.au; 2 Ampcontrol Pty Ltd., Tomago NSW 2322, Australia; steve.mitchell@ampcontrolgroup.com

**Keywords:** partial discharge detection, UHF sensors, power system equipment, antenna, insulation condition monitoring

## Abstract

Condition monitoring of an operating apparatus is essential for lifespan assessment and maintenance planning in a power system. Electrical insulation is a critical aspect to be monitored, since it is susceptible to failure under high electrical stress. To avoid unexpected breakdowns, the level of partial discharge (PD) activity should be continuously monitored because PD occurrence can accelerate the aging process of insulation in high voltage equipment and result in catastrophic failure if the associated defects are not treated at an early stage. For on-site PD detection, the ultra-high frequency (UHF) method was employed in the field and showed its effectiveness as a detection technique. The main advantage of the UHF method is its immunity to external electromagnetic interference with a high signal-to-noise ratio, which is necessary for on-site monitoring. Considering the detection process, sensors play a critical role in capturing signals from PD sources and transmitting them onto the measurement system. In this paper, UHF sensors applied in PD detection were comprehensively reviewed. In particular, for power transformers, the effects of the physical structure on UHF signals and practical applications of UHF sensors including PD localization techniques were discussed. The aim of this review was to present state-of-the-art UHF sensors in PD detection and facilitate future improvements in the UHF method.

## 1. Introduction

The reliability of the electrical insulation within high voltage equipment can significantly affect the lifespan of the apparatus given the working conditions and stability of a power system’s operation. Insulation deterioration can occur gradually and continuously even under normal operation. Thus, maintenance work should be employed in a timely manner to prevent catastrophic failures. Currently, condition monitoring systems employed in power equipment operations aim to detect early indicators before a fault can occur and provide guidance on the fault [1,2]. Partial discharge is a detectable fault commonly occurring in an insulation system within power installations. This discharge can result in gradual insulation deterioration and aging acceleration in the main insulation, and consequently, could result in full insulation breakdown if not appropriately treated at an early stage [3]. Thus, partial discharge (PD) detection is an important tool for insulation health diagnostics in equipment maintenance. Regarding PD detection, a range of methods have been developed and applied to insulation testing including the electrical method, based on the IEC 60270 standard, along with other non-conventional methods, to achieve satisfactory monitoring capability. Non-conventional methods is a reference to PD detection based on the physical phenomena accompanied with the PD events such as electromagnetic (EM) waves, acoustic pressure waves, chemical by-products, etc. [4,5,6,7]. Given the potential limitations of the IEC 60270 method for on-site testing (which is sensitive to external noise and interference); the ultra-high frequency (UHF) method has drawn researchers’ attention over the past few decades. Partial discharge detection based on UHF sensors is advantageous for on-site insulation monitoring due to its immunity to external EM interference, and is hence more suitable for on-site testing than conventional methods [8,9]. The rise time of a discharge’s impulse can be shorter than 1 ns such as in oil insulation [10]. Such a short impulse can emit EM waves with frequency components in the UHF range, i.e., from 300 MHz to 3 GHz. This method is implemented using the UHF sensor (antenna) to capture the EM waves emitted from the PD source and was developed for gas-insulated switchgear (GIS) in 1988 [11] and then applied to power transformers and cables in 1997 [12] and 1998 [13], respectively.

To date, several review works related to PD detection based on UHF measurement have been conducted in recent years in terms of signal processing [3], localization [14,15], and pattern recognition [3]. However, few types of research have drawn on the systematic review of UHF sensors applied in PD diagnosis. In this regard, state-of-the-art UHF sensors applied in PD detection will be comprehensively presented in this review. This paper takes the form of five sections, including this introductory section. It will then go on to the UHF sensor design and performance evaluation in Section 2. The third section is concerned with the effects of the structure of the power transformer on UHF detection. Section 4 presents the application of UHF sensors in PD locating techniques. Finally, the last section provides a summary and includes a discussion on the implications of the findings with respect to future research into this area.

## 2. Partial Discharge Detection Methods

As mentioned in Section 1, PD detection methods are substantially dependent on the products generated in the discharge process. Table 1 shows various PD detection methods related to the physical properties including: electrical discharge current impulse, by-products from chemical reactions, acoustic emission (pressure waves), and EM waves radiated at various frequency ranges (e.g., VHF, UHF, light); the corresponding methods and applied sensors are also given.

Among the detection methods, the UHF method has, as its leading merits, its immunity to external electromagnetic interference and the high signal-to-noise ratio with high detection sensitivity [16]. Noise at a lower frequency range (below 200 MHz), such as the surrounding continuous white noise within a substation, can be prevented. For equipment with a steel tank or shell, external electromagnetic interference from surrounding corona discharge can be mitigated effectively due to the shielding effect of the metallic structure. However, if the sensor is mounted externally, EM radiation from telecommunication systems such as TV and radio broadcasts can affect the accuracy of the UHF method at on-site PD monitoring locations [17,18]. Thus, incorrect monitoring information may occur due to these interferences. Judd et al. [19] state that communication systems are not a serious concern since these signals continuously exist, whilst a PD event is an impulse signal, which is possible to discriminate and extract from the polluted signal. Besides, localization can be achieved. This is another reason to implement the UHF method in PD monitoring systems since the severity of the PD activity is not only related to the amplitude of the discharge, but also dependent on the location. The position information can provide targeted guidance for further maintenance work.

Although UHF has good performance in terms of its detection sensitivity, the primary concern with regards to its application is the lack of a reliable calibration process. Calibration is required to develop the quantitative relationship between the discharge severity and the magnitude of the UHF signal. This issue will be discussed in more detail in Section 3.4. Another challenge for implementation of the UHF method is that the measurement system requires a high sampling rate and the hardware costs can be high for the processing and storage of such significant amounts of data [20,21]. A solution was proposed in [22] where a UHF–HF converter was employed to transform the UHF pulse to a lower frequency and process the signal using a measurement system which was valid for HF analysis. This can downscale the dataset and has little influence on the accuracy of the detection. In light of this, UHF sensors are commonly used in conjunction with other detective sensors in a complementary manner. Examples are coupling capacitors, HFCTs, and acoustic sensors [22].

## 3. UHF Sensors in Partial Discharge Detection

**Figure 1:**
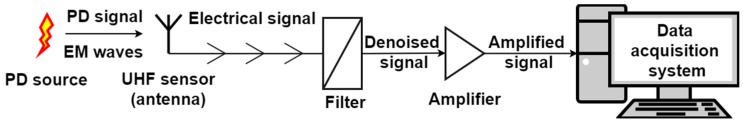
A general process of ultra-high frequency (UHF) measurement.

In engineering applications, UHF sensors have been widely used to detect defects such as cracks in physical structures [23], displacement and tilt detection in wireless radio frequency identification systems [24], and partial discharge measurements in high voltage engineering [17]. These applications are practically feasible because the transient process of these defects have very short rise times, which results in induced frequency components in the UHF range. As can be seen in Figure 1, a UHF sensor plays a significant role in UHF PD measurement because the initial step of PD measurement is to acquire electromagnetic signals using these devices for further signal processing. To this end, the performance of the sensors will dramatically influence the accuracy and sensitivity of the PD detection system. Considering the nature of the detected signal, UHF sensors can be regarded as antennas since these sensors are required to receive the induced EM waves from the PD source. An antenna is defined by the IEEE Antennas and Propagation Society in [25] as “that part of a transmitting or receiving system that is designed to radiate or to receive electromagnetic waves.” Such detection devices are used as energy conversion devices to convert the coupling EM signal produced by the PD source to a current or voltage signal.

### 3.1. Sensor Classification and Installation

**Figure 2:**
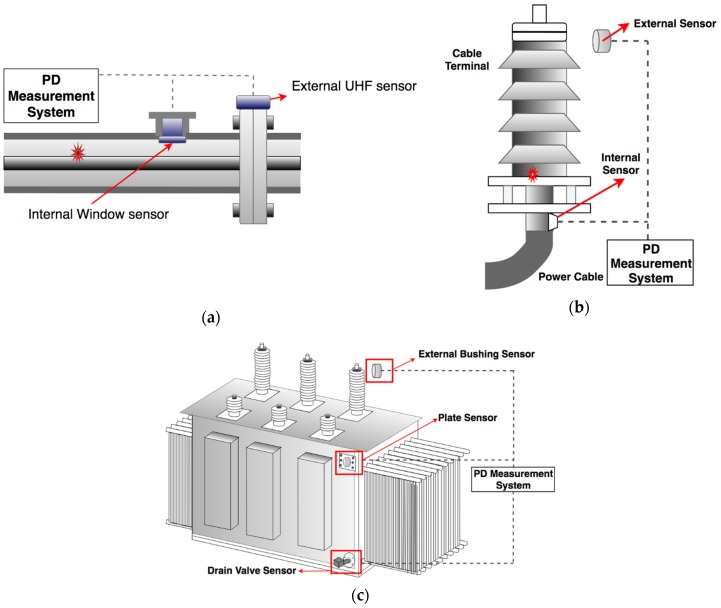
Installing locations for UHF sensors: (**a**) GIS; (**b**) cable terminal; (**c**) power transformer.

**Figure 3:**
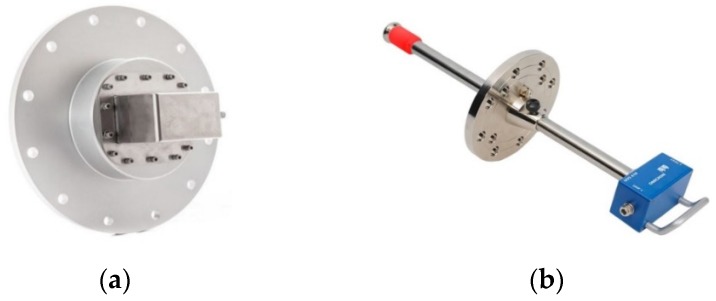
UHF sensors applied in a power transformer: (**a**) internal UHF sensor; (**b**) Omicron UVS 610 UHF valve sensor [28].

The UHF method for PD measurement has been widely applied in power equipment including power transformers, cables, rotating machines, switchgear, and gas-insulated substations [22,26,27], with the UHF sensor arrangement being application specific. Figure 2 presents some installation examples of UHF sensors in different equipment. Sensors are generally classified as either internal or external depending on their placement relative to the equipment. Figure 3 also highlights the configuration differences between an internal UHF sensor and a valve UHF sensor when fitted to a power transformer. With regards to the internal sensor, the antenna mounted on a metallic shell can be varied by the designer in order to improve the detection performance. This is in contrast to valve sensors which are commonly based on a monopole antenna design. The insertion depth of a valve sensor can be adjusted to achieve the desired frequency response. More detail will be given in Section 3.2.1.

As the UHF method has been around for several decades, a series of commercial UHF sensors which have been well-designed for on-site PD monitoring, are listed and compared in Table 2. These commercial products are more likely to have higher compatibility with the high voltage equipment installation and standardized testing procedures when compared with those that have been proposed for experimental research. However, sensor performance is typically a performance tradeoff dependent upon its application and conditions in the field.

Gas-insulated switchgear is widely employed in modern power substation installations due to the high operating reliability and the small occupied area. In GIS, the sensors can be mounted both internally and externally. Internal sensors are directly attached on the inner surface of the tank and couple the EM signals through barriers. The most common installation method is an external installation approach which includes a windowed coupler and a barrier coupler at different positions. This method is unlikely to affect the continuous operation [34]. Besides the traditional installation, Li et al. [35] found that the metallic rod-like inner shield case, which is an intrinsic structure in the gas insulated inverted current transformer (SIICT), can be seen as a monopole antenna and used for picking up UHF signals inside the SIICT. This provides a convenient solution to the challenge of mounting additional sensors in the equipment.

Unlike the GIS apparatus, sensor installation is a challenge for the UHF method when used in power cable and power transformer applications. This is because there are no dedicated apertures for sensor installation, and the structure for operating equipment cannot be easily modified without a power outage. For cables, it is not ideal to place a UHF sensor external to the cable since the conductive sheath layer can effectively shield the electromagnetic waves, which provides a challenge to PD detection. Therefore, the UHF method is used to monitor power cable terminals by coupling the sensors with the inner accessories (or the cable itself) so that the weak discharge can be extracted without external EM interference. For a power transformer, sensors can be inserted through the oil drain valve, a dielectric window [36] or placed externally to receive the EM wave as shown in Figure 2. To install the sensors through the oil drain valve, the structure of the oil valve should be taken into consideration. Valves without a proper straight opening, such as diaphragm or butterfly valve, cannot fit such sensors [37]. The number of available oil drain valves is another limitation for UHF installation. For a real transformer, there are generally no more than three oil valves which could be utilized for antenna installation [38]. For large-scale power transformers, UHF signals can leak through the non-metallic insulating gaps at the edges of the transformer tank [39] and bushing taps [16,40] where the external UHF sensors can detect the inner PD signal without a complicated installation process. The sensitivity for such an approach may be lower than via an internally detected method, but this method is simpler for on-site sensor installation [40].

Based on the above discussion, sensor installation is an important factor in PD detection. Safety issues may arise if the sensor is too close to high potential parts and the receiving signal could be weak if the sensor is too far from the defect. Also, size limitation imposes difficulties on the sensor design because specific sensor performance may need to be improved by increasing the geometrical dimension [41]. Thus, compromises are made to balance the relationship between the antenna performance and the size. In this regard, external sensors are easily installed with higher flexibility; however, the signal received by the antenna could be weak due to the attenuation along the distance between the PD source and sensors. Also, the effect of the surrounding grounded boundary including the grounded drain valve [42], the relative position of the transformer tank around the sensor and its insertion depth, has been demonstrated to influence the results in previous research [16,37]. However, some of these results were based upon software-based simulation and require experimental verification. Compared to external sensors, internal sensors have a much greater signal-to-noise ratio because they are much closer to the discharge source. Higher sensitivity and anti-interference is the main advantage of an internal setup, but internal antennas should typically only be placed in the tank during the manufacturing process, rather than during operation [37,43]. Also, the internal sensor should not alter the inner operating conditions such as the electric field distribution or introduce potential electrical defects into the equipment. For example, the design proposed in [44] is fabricated using metal with a sharp point and a folded corner; this design may not be appropriate as an internal sensor since it will adversely affect the field distribution and potentially become a discharge source. As mentioned in [37], a dielectric window integrated with a welded ring should be mounted during the production and a plate UHF sensor can be installed and swapped conveniently on-site.

With the development of wireless communication technology, wireless sensors provide high flexibility for sensor installation and data transmission [45,46]. However, these techniques are more likely to be susceptible to the surrounding interference in signal transmission and propagation at the local substation. Moreover, in [47], a moving robot equipped an antenna with omni-directional directivity was proposed for substation inspection. This can provide a higher level of automation in equipment maintenance.

### 3.2. Sensor Design

There are two steps commonly involved in the UHF sensor design process including simulation by software and practical fabrication. Software for electromagnetic simulation, including CST Microwave Studio [41,48,49], Ansys HFSS [50], and MAGNA/TDM [44], can be used for UHF sensor modeling and far-field EM characteristic simulation. In this section, several typical UHF sensors will be introduced and a comparison of proposed UHF sensors in recent literature will be made in terms of their performance.

#### 3.2.1. Monopole Antennas

**Figure 4:**
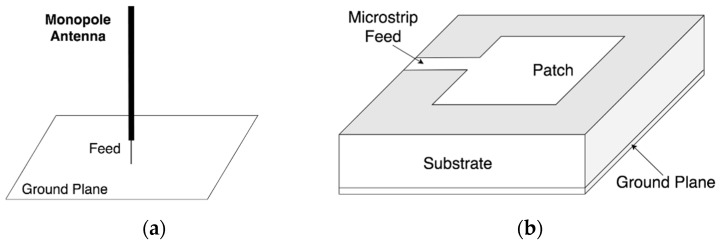
Typical antenna structure: (**a**) monopole antenna; (**b**) microstrip antenna.

Monopole antennas are widely used because of the simple structure which is shown in Figure 4a, good radiation pattern, and suitable size. However, the working bandwidth of general monopole antennas is narrow; this will lead to information loss. The conical antenna is a monopole antenna which has been commercially used in UHF PD signal detection as shown in Figure 3b [16,28,51]. The Omicron UVS 610 UHF sensor can be equipped through both DN-50 and DN-80 standard oil drain valves.

#### 3.2.2. Micro-Strip Antenna

The microstrip antenna is also proposed in the literature for PD detection in the UHF domain [43,52,53,54,55]. The basic structure of the microstrip antenna is shown in Figure 4b. This type of sensor can be a feasible alternative to the commercial UHF probes due to the main advantages of the microstrip antenna, which are small thickness, low mass, low fabricated cost, and small volume [56]. However, the primary limitations of such an antenna are narrow bandwidth, along with the high ohmic and dielectric loss. 

#### 3.2.3. Fractal Antenna

**Figure 5:**
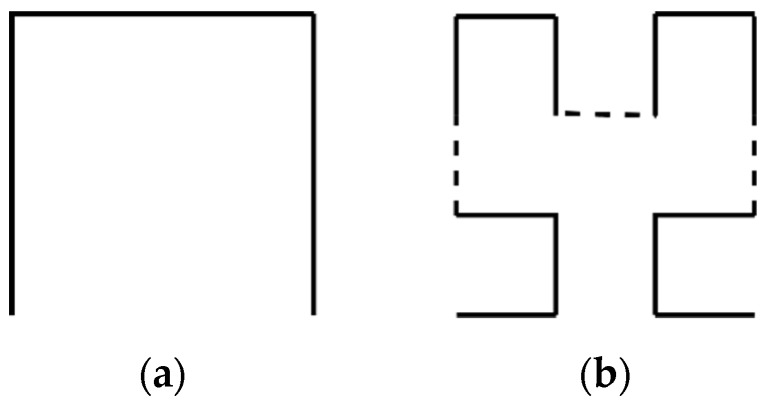

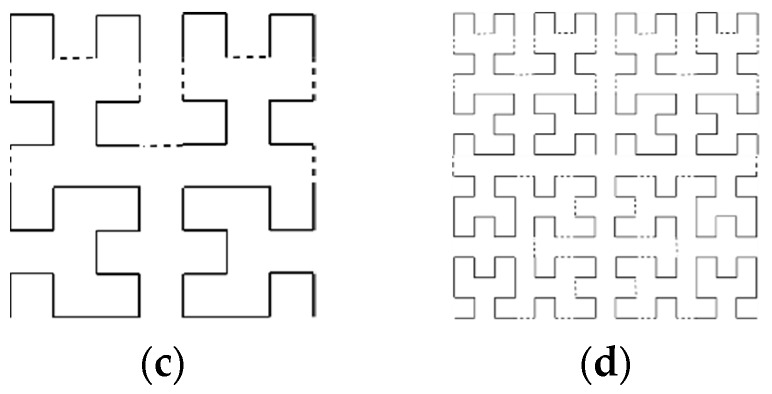
Iterative orders of Hilbert fractal curve: (**a**) first; (**b**) second; (**c**) third; (**d**) Forth.

A fractal antenna is one type of microstrip antenna with distinctive performance in UHF signal coupling and can offer miniaturization and wide bandwidth. Wang et al. [52] designed a Minkowski fractal antenna and found that the order of the fractal curve can significantly affect the performance of the antenna. The Hilbert fractal antenna was proposed for PD detection in [56,57,58], and it was found that the fourth-order Hilbert fractal structure shown in Figure 5d is appropriate for multiple-resonance filter design. Meander fractal structure was designed in [59], and the result shows that the gain regarding the radiation pattern increases significantly with higher fractal order.

#### 3.2.4. Ultra-Wideband Antenna

Ultra-wideband (UWB) measurement can be particularly useful for PD analysis of oil-filled power transformers and in [55], Yang et al. proposed a U-shaped UWB antenna for PD detection in power switchgear. As a wide range of frequency components can be detected using this type of antenna, PD recognition can be applied with higher accuracy when used in conjunction with frequency spectrum analysis. 

### 3.3. Sensor Optimization

Various antennas have been introduced in Table 3; these antennas have relative advantages and limitations in function, structure, and operating characteristics. To improve the performance of the antenna, many researchers have examined the optimization of antenna parameters by both simulation and experimental measurements to get a higher level of detection accuracy and sensitivity. Parameters such as S-parameters, voltage stand wave ratio, and input impedance can be measured using a vector network analyzer for comparison between the simulation result and fabricated products [16,37,42]. Other performance-related specifications such as surface current distribution and directivity (gain in E-plane and H-plane) can be observed via simulation using CST Microwave Studio. In this section, parameters which can represent the performance of the antenna will be introduced based on the IEEE standard on antenna design [25] and the parametric optimization techniques proposed in recent research are examined.

#### 3.3.1. Directivity (Radiation Pattern)

**Figure 6:**
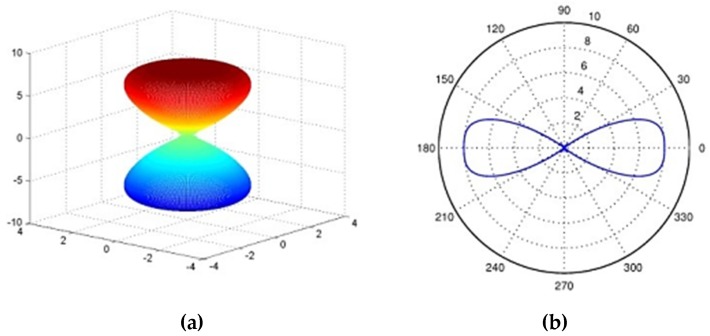
Illustrations of a sensor radiation pattern (**a**) 3D dimension (**b**) 2D dimension.

The radiation pattern represents the capability of the antenna in receiving and transmitting signals at a certain direction. The parameter describing such a characteristic is the power gain. The radiation patterns are often illustrated using 2D or 3D figures to show the far field features, namely the value of power gain at different frequencies as shown in Figure 6.

The direction with the maximum gain should be towards where, statistically, PD frequently occurs. Those locations which are highly susceptible to PD are more likely to cause insulation breakdown over long-term operations. The radiation pattern can be classified into two primary forms: omnidirectional and unidirectional, and for 3D directivity representation, both radiation patterns in x-y and y-z planes should be considered and analyzed. Yang et al. [55] believed it is advantageous that the antenna can receive EM signals from all directions and so proposed a novel UWB antenna with omnidirectional capability. By contrast, Lee et al. [50] states that a unidirectional pattern can improve the sensitivity towards a specific direction since they can detect signals directionally. This feature is taken into account and implemented in [30]; the direction of the discharge device can be roughly estimated by changing the orientation of the sensor. For unidirectional radiation enhancement, a cavity-back structure and superstrate are proposed in [50] for directivity optimization purposes.

#### 3.3.2. Size

To evaluate the size of the antenna, the electrical size [50] is more meaningful in the analysis rather than the geometrical size. For size comparison, the latter will simply take the longest physical side, whereas the electrical size represents the ratio of the physical size to the wavelength corresponding to the lowest working frequency. 

#### 3.3.3. Gain

This parameter is defined as “the ratio of the radiation intensity in a given direction to the radiation intensity that would be produced if the power accepted by the antenna were isotopically radiated” [25]. It is commonly measured in dB and calculated by

(2) $$G_{dB}=10logG=10log(D\eta )$$

where *D* and *η* denote the directional coefficient and antenna efficiency respectively. The value of the gain is expected to be high in order to effectively discriminate the PD activity from the external interference in field testing. In some sensor designs, in order to accommodate other requirements such as the geometrical size and radiation pattern, the gain level may be sacrificed. However, this issue can be solved by using pre-amplifiers.

#### 3.3.4. Input Impedance

(3) $$Z_{in}=Z_{c}\times \frac{1+\Gamma }{1-\Gamma }$$

where $Z_{in}$ is the total input impedance of the antenna and $Z_{c}$ is the characteristic impedance of the transmission line. In this equation, Γ represents the reflection coefficient:

(4) $$\Gamma =\frac{Amplitude\;of\;the\;reflected\;wave}{Amplitude\;of\;the\;incident\;wave}$$

For the purpose of electrical matching with the connecting coaxial cable of the measurement system, antennas are commonly designed with an impedance of 50 Ω or 75 Ω. Generally, the input impedance is a complex number with both resistive and reactive components. It may be necessary to decrease the value of the reactive component to better match the antenna and the feedline. In some cases, a balun is needed to transform the impedance as part of the antenna design [50]. However, this can increase the complexity of the structure and fabrication process. The parameter is also affected by the surrounding environment of the sensors, especially the conductive material with certain potential or earthing [56]. The parameter associated with evaluating the level of impedance mismatch is described in Section 3.3.5.

#### 3.3.5. Impedance Matching Parameters

To evaluate impedance matching, the voltage stand wave ratio (VSWR) and return loss (RL) are commonly used in antenna design. The equations to calculate these two parameters are:

(5) $$VSWR=\frac{1+\left|\Gamma \right|}{1-\left|\Gamma \right|}$$

(6) RL=20log|Γ|

The VSWR represents the level of the impedance mismatch between the antenna and the feed line transmitting the radio frequency signals. When the EM wave propagates from one medium to another, some reflection will occur due to the mismatch between the materials. The value of VSWR ranges from 1 to infinity. For UHF sensor design, the VSWR should be lower than 2 at the working frequency range; in other words, the power reflected should be less than 10%. 

**Figure 7:**
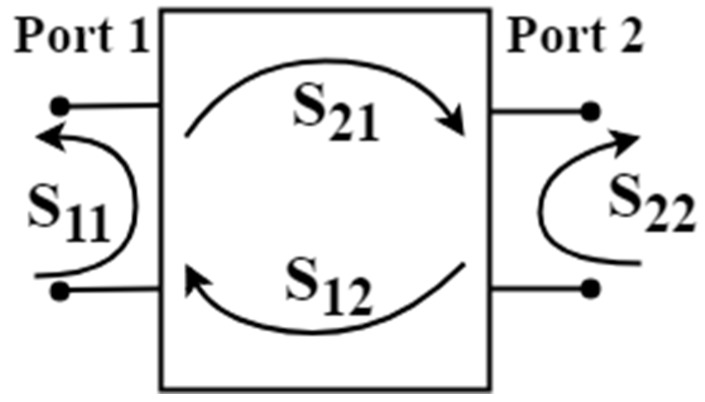
S-parameter in two-port network.

In addition, the S11 parameter, namely the return loss, can also be used to describe the reflection due to impedance mismatch as shown in Figure 7. The antenna and the feed line correspond to port 1 and port 2, while the proportion of reflective power back to the source at port 1 should be reduced in the design process. The acceptable level of the return loss is generally below 0.1 (−20 dB).

#### 3.3.6. Frequency Bandwidth

The frequency bandwidth of the antenna is defined by equation:

(7) $$B=\frac{f_{max}-f_{min}}{f_{0}}\times 100\%$$

where $f_{max}$ and $f_{min}$ denote the upper and lower cutoff frequencies respectively, and $f_{0}$ is the center frequency of the passband. According to the antenna theory, antennas can be classified into three types considering the measurement bandwidth as shown in Table 4.

In UHF measurement, both wideband and narrowband strategies are used for different purposes. Some believe that a wide bandwidth can cause a higher external noise level and distortion with respect to its influence on pattern recognition. In [51], the measured frequency range was only 70 MHz for UHF pattern recognition but the authors found distinguished features of different discharge patterns can be extracted in such a narrow bandwidth. On the other hand, Sinaga et al. [41] and Lee et al. [41,50] argued that narrow bandwidth will lead to loss of information in the frequency domain and be considerably more susceptible to UHF signal distortion. When analyzing the PD activities in the frequency domain, the selected range should be considered based on the tank dimensions [69]. In [56], researchers consider the external EM interference from radio, TV, and telecommunications can be alleviated by multi-band (multi-resonance) frequency response design. Specifically, the sensors can be designed to have higher sensitivity at the frequency range generated by PD activities and effectively filter out the unwanted disturbance.

A broad bandwidth is preferred in PD sensor design for several reasons. Firstly, the signal captured by the sensor can provide more information in the frequency analysis with a broader detection range. Furthermore, a signal which contains significantly more frequency components can represent the PD pulse wave shape more accurately [41]. Also, the bandwidth should be such that its operating frequency range will maintain the parameters of the sensor within a required range including the impedance bandwidth, VSWR bandwidth, and gain bandwidth. During the design process, it is critical to optimize the antenna bandwidth based on the three ranges above. Contributions such as [59] and [50] merely take the impedance bandwidth and the S11 bandwidth, respectively, into the sensor design process, without providing evidence that the other two parameters meet the requirements. For microstrip antennas, the antenna performance was highly dependent on the metallic patch and the dielectric substrate. The measurement bandwidth of the antenna can be widened by using a material with low permittivity and increasing the thickness of the substrate. A notable wedge-shaped substrate proposed in [70] gave an approximately two-fold increase in bandwidth compared to a rectangular substrate. However, this may increase the complexity in fabrication.

#### 3.3.7. Surface Current Distribution

For better performance of the microstrip antenna, the surface current distribution should be concentrated along the microstrip feed line and the excitation port rather than the shaped patch [55]. This is considered for both thermal stress and the loss of the sensors because the nature of the dielectric substrate may be affected by the temperature.

#### 3.3.8. Operating Environment and Reliability

The UHF signals are received via antennas and their radiation pattern will be significantly affected by the temperature of the surrounding area [71]; an example would be the influence of temperature variation associated with the insulation oil in an operating power transformer. Moreover, humidity may also affect performance. In addition, it is essential that UHF sensors are designed for a long working life with low incidence of fault since replacement may require the shutdown of an installation and resulting power outage.

### 3.4. Sensitivity Check and Calibration

Sensors with high sensitivity enable PDs that occur deep inside the insulation to be detected, in other words, very weak EM signals resulting from attenuation and reflection can be adequately captured by the antenna. Compared to the conventional electrical method in PD measurement, a weakness of the UHF method is its lack of a suitable calibration method or a standard sensitivity test [16,72]. Therefore, the UHF method has not been considered as standardized testing as yet [37]. The relationship between the UHF and IEC 60270 methods has attracted significant research interest since the electrical method has already been standardized and can quantify the relationship between apparent discharge and true discharge. The idea of replacing the conventional electrical measurement approach with the UHF method during the induced voltage test has raised concerns in terms of its feasibility and accuracy. It may be difficult to find a reasonable correlation from the UHF signal magnitude to the amount of charge in pC; however, if this relationship can be achieved, the sensitivity verification can be done on site via the UHF approach which overcomes the on-line performance issues associated with the IEC 60270 method [36]. Given that the UHF measurement in a power transformer is affected by many factors, including detection frequency range, defect type, physical internal barriers along with the characteristics of applied UHF sensor, the calibration is more problematic with a higher degree of uncertainty as compared to conventional measurement [73]. Therefore, the validity of the UHF method to estimate the severity of the PD activity is an issue requiring further investigations.

For a sensitivity check, a pulse generator is required [74], which can emit pulses with adjustable magnitude at a constant repetition rate. An Omicron UPG 620 Pulse Generator can be used for UHF calibration/measurement. The peak output voltage is ranged from 0.5–60 V for both positive and negative values and the rise time can be less than 200 ps [31]. The checking process can be conducted on a transformer with a UHF calibrator or an artificial PD defect, utilizing antennas as both signal emitter and signal receiver. The defect can be modelled using a spark generator or an artificial PD source utilizing an electrode system placed within a transformer’s dielectric window [75].

The attenuation of UHF signals is discussed in [72], several identical monopole antennas are mounted at different positions in the transformer to receive the impulse signal. A UHF PD probe is used to emit the signal generated by the UHF calibrator at a fixed position. Consecutive impulses with constant magnitude are produced and the attenuated effect is analyzed in terms of the receiving signal magnitude at each position.

In [76], a linear relationship was proposed between the magnitude of the UHF signal and the apparent charge. This is the earliest development in terms of bridging these two methods even though the research is based on GIS. Martinez-Tatifa et al. [77] attempted to find a quantitative relationship between the IEC 60270 method and the radio frequency (RF) method regarding the peak magnitude and energy in both the high frequency (HF, 3–30 MHz) and UHF domains in a power transformer. A corona discharge model (needle-plate) and a surface discharge model were used in this experiment to produce PD signals. Both HF and UHF signals were captured for analysis in this research; the HF signal was detected using HFCT and the UHF signal was detected using two different antennas (i.e., monopole and Vivaldi). The amplitude and the energy of the HF and UHF PD pulses were measured and calculated, and fitting functions were proposed to fit the data. The fitting performance was evaluated using the statistical parameter Pearson’s coefficient. According to the fitting results, a large proportion of the data points were scattered with a low Pearson’s coefficient. Three sets of data may have statistical correlations regarding their high fitting coefficient: the UHF (400–800 MHz) signal energy relative to the HF signal energy in corona and surface discharge by a linear fitting function, and the HF signal peak voltage versus the UHF signal energy by an exponential curve fitting [77].

For sensitivity measurement, “Effective length” was defined to evaluate the sensitivity of the antenna. This parameter was measured in millimeters [36,74]:

(8) $$Effective\;Length\;\left(L_{e}\right)=\frac{1}{Antenna\;Factor\left(AF\right)}=\frac{Voltage\;at\;the\;antenna\;terminals\;\left(V\right)}{Incident\;electrical\;field\;strength\;\left(V/mm\right)}$$

**Figure 8:**
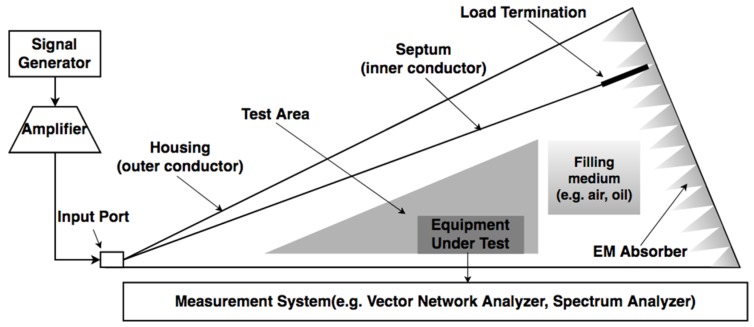
GTEM cell for UHF sensor sensitivity testing.

It can be noted that the sensitivity of the antenna increases with higher effective length value. Based on the measurement requirement, the sensitivity of the UHF sensor is at least 6 mm, i.e., the received voltage of the antenna at the specific frequency is 6 mV (RMS) for an incident electric field of 1 mV/mm (RMS) [78]. In other words, the incidence field is part of a propagation EM wave generated by the PD source and the effectiveness of the antenna is shown as the magnitude of the receiving signal. To investigate $L_{e}$ of the antenna, the experiment can be conducted using the gigahertz transverse electromagnetic (GTEM) calibration system which is shown in Figure 8. This set of devices can generate a uniform electric field without internal reflection or external interference at a wide range of frequencies. Ishak et al. [78] investigated the antenna performance by both finite element time domain (FDTD) simulation and experiments using the GTEM cell, and the results show good agreement. This provides a novel way for UHF sensor design and performance verification. It is preferred that the experiment is conducted using an oil-filled GTEM cell to emulate the condition inside the transformer rather than an air-filled cell [37,74], because the permittivity of the inner medium affects the propagation velocity and the frequency components excited in the cell [79].

## 4. Effect on UHF Signal Propagation in a Power Transformer

The power transformer is one of the most crucial and expensive pieces of equipment in a power system network. Transformer failures result in high replacement costs and can even lead to catastrophic consequences within an energy utility if not actioned expediently. According to recent surveys on the failure mode and retirement rate of power transformers [80,81], the statistics indicate that winding and insulation related issues are the major cause of transformer failure. Therefore, the operating reliability of windings and insulation should be enhanced for both economic and safety reasons. To monitor the health of these components, the PD level is an effective indicator for diagnostic and the UHF PD detection method is capable as an on-site monitoring tool.

Compared with other electrical apparatus, power transformers have a complex structural configuration with inner winding-core structures and bushings on the top. This can lead to reflection, scattering and attenuation of EM waves emitted from the PD source, including the UHF components [82]. Thus, it is useful to analyze the potential effects of these structures on such a method. From previous literature, a series of PD experiments were employed in an oil-filled glass container [83,84] and air-filled metallic grounding tank as a transformer model without internal barriers [85,86] for simplicity. The results may differ significantly if repeating the tests on a real transformer and differences must be assumed. Moreover, there is some literature regarding conducting simulation [42] or experiment [16] based on an air-filled tank model with results considerably different when compared to analysis on an oil-immersed transformer. Characteristics of the induced EM wave propagation have been analyzed by several researchers using CST Microwave Studio for simulation [10,47,87] and in operating power transformers in field study [88,89]. Furthermore, there is an increasing number of researchers conducting simulation by means of the FDTD method to analyze the characteristics of the PD induced EM wave: frequency response analysis of the UHF sensor design [90,91,92] and characteristics of EM wave propagation [42,48,82,93]. This approach can effectively model the structure of the transformer and simulate the signal strength in the electromagnetic domain.

### 4.1. Effect of the Insulation Material

The main insulation in an oil-type transformer is oil/paper insulation, and the EM waves generated by the PD source propagate primarily through the transformer oil. The propagation velocity of the signal can be determined by:

(9) v=1με

where μ and ε represent, respectively, the permeability and the permittivity of the insulation medium. The relative permittivity of the transformer oil is commonly used as 2.3 [94]. The velocity can also be found by experimental testing if the parameters are not available, the speed of EM wave is found as 2 × 10^8^ m/s in [95], which is two thirds the speed of light. Although the paper insulation presents a solid barrier along the propagation path, the detection sensitivity is not affected significantly with negligible scattering and attenuation [96].

### 4.2. Effect of the Transformer Tank

The effect of the transformer tank on UHF measurement is mainly represented as the grounding effect, shielding effect [97] and the enclosure effect [98]. 

The grounding tank can affect the surrounding field distribution of the UHF sensors and hence the sensor performance [96,97]. Jahangir et al. [16] found that the position of the dielectric window for the UHF probe installation can affect the radiation pattern and reflection loss of the antenna, respectively. Improved radiation pattern and non-effective lobes are shown when conical antenna is mounted on the dielectric window that is located in the middle of the transformer wall, whereas the return loss of the antenna is not significantly affected. However, this finding is based on software simulation in CST studio, which is not validated via experiment. Moreover, the transformer tank wall is set to be a perfect conductor for simulation, which may result in minor errors when taking the material permeability into account.

Furthermore, insertion depth is another factor which should be considered during sensor installation, especially for oil drain valve sensors. Jahangir et al. [16] examined how the effect of insertion depth on the antenna performance is potentially dependent upon the detecting frequency components. Impedance matching characteristic and radiation patterns can be influenced at lower frequency ranges (e.g., <700 MHz) and higher frequency ranges (e.g., >1500 MHz) [16]. In this regard, they believe that increased insertion depth has a positive impact on the performance of the antenna. Siegel et al. [37] noted that the inserted position where the antenna is plugged into the tank offers higher sensitivity by comparing the antenna factor which is mentioned in Section 3.4. This has been demonstrated experimentally in both oil-filled transformer tanks and GTEM cells with a UHF drain valve sensor.

As for the shielding effect, Robles et al. [97] studied this by measuring the internal PD events with antennas inside and outside the transformer tank simultaneously. The signal magnitude shows high level of attenuation in the time-domain and dissimilarity in the frequency response. This can assist to discriminate the PD when it occurs both internally and externally and improve the detection accuracy in a field study.

In addition, the enclosed structure of the tank can be treated as a rectangular resonant cavity. The EM wave generated from the PD source may be reflected and attenuated before arriving at the UHF sensors. The resonant frequency which can be calculated by Equation (9) needs to be considered when analyzing the features of the UHF signal propagation [99].

(10) $$f_{r}=\frac{c_{0}}{2\sqrt{\varepsilon_{r}}}\sqrt{{\left(\frac{m}{h}\right)}^{2}+{\left(\frac{n}{l}\right)}^{2}+{\left(\frac{p}{w}\right)}^{2}}$$

where, $c_{0}$ is the propagation velocity of light; $\varepsilon_{r}$ is the relative permittivity of the inner insulation oil ($\varepsilon_{r}$ = 2.3); *h*, *l*, *w* are the height, length and width of the transformer tank inner dimensions; *m*, *n*, *p* are the eigenvalues of the propagation eigenfunctions. Therefore, the frequency spectrum of the UHF signal received by the antenna would be dramatically affected by the geometrical features of the transformer [97]. The frequency components at resonant mode can be identified and detected inside the transformer, while the tank can be seen as a low-pass filter resulting in frequency components above a threshold not being captured outside the tank. Although the reflection of UHF signals may superimpose on the waveforms, Tang et al. [96] state that the effect can be mitigated if the transformer dimensions are large enough.

### 4.3. Effect of Internal Barriers

The primary effect caused by the internal structure, including the transformer winding and the core, is that these are likely to influence the propagation of the EM waves [10,42,82]. The effects can be simulated by a finite differential method of time domain (FDTD) [42,57,82,93] and the finite integration technique (FIT) algorithms [100]. The FDTD method has the advantage of high versatility, accuracy, and robustness [101]. Electromagnetic simulation software such as CST Microwave Studio can be used to simulate the propagation of EM waves [101], but Zanjani et al. [48] highlighted that only small-scale transformer models can be simulated by such software due to memory limits of the computer and they proposed the FDTD method programmed in a C++ environment. Analysis of the simulation results of the E field showed that the iron core affects the time of arrival [42] and received signal magnitude more significantly [82] than the winding. While Li et al. [57] simulated the effect of the barriers within a very large-scale transformer winding, and the result showed that the EM signal cannot propagate through the winding if the winding scale is large enough. However, this issue can be mitigated by optimizing the sensor placement. In [10,102], the effect of various types of transformer core in terms of the iron packet core, the steel cylindrical shell core and the wooden cylindrical shell core is investigated by simulation. Furthermore, Azirani et al. [69] investigated the effect of the transformer tank on the receiving UHF signal by experimental study. The experiment result is consistent with the simulation result in [16], the grounding tank configuration can affect the scattering parameters of the UHF antenna including the reflection and transmission coefficient. Furthermore, these scattering coefficients can influence the measurable frequency. Thus, the measurable frequency range is varied depending on the geometry of the test object, and this should be considered in experiment design.

Also, Tang et al. [96] showed the effect of the core and winding on the time of arrival (TOA) of the UHF signal by experimental studies. Real iron core and transformer windings are used as the internal barrier, and the result is consistent with the simulation. The core structure has a definite impact on the TOA delay and signal attenuation, while the transformer winding does not. To reduce the error of TOA caused by the iron core, a diamond-shaped sensor array with four monopole antennas is proposed, and the accuracy of TOA measurement is improved [96]. Coenen et al. [38] state that the influence of these barriers presenting along the propagating path can be eliminated using signal processing tools.

### 4.4. Effect of Bushing Installation

Due to the presence of non-metallic bushing, the transformer tank is no longer an enclosed Faraday cage, and shielding against external EM interference will be negatively influenced [37]. Jahangir et al. found that despite the shielding provided by the tank, corona interference from adjacent equipment cannot be entirely removed due to the presence of the high voltage bushings on the top of the transformer [16].

### 4.5. Effect of Other Structure

The effect of the grounding drain valve on VSWR was investigated by simulation [42]. The result shows that the influence was not apparent between whether the drain exists or not. Fauzan et al. [45] analyzed the effect of capturing the signal via the BNC terminal, which was often ignored in the experiment.

## 5. Partial Discharge Localization by UHF Sensor Array

**Figure 9:**
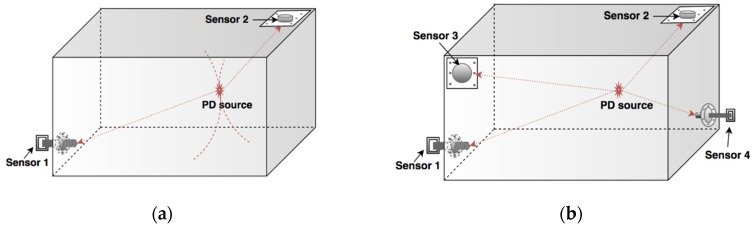
UHF sensor arrangement for PD localization (**a**) with two sensors (**b**) with four sensors.

With appropriate positioning of the UHF sensors, locating the PD sources can be achieved by comparing between the signals captured from different sensor positions. When a PD occurs, the discharge source will radiate EM waves, and signals are received by the antenna with different arrival time as shown in Figure 9b. With the sensor array technique, the TOA determination can be improved by using the correlation coefficient of signals captured from different sensors [96]. Considering the EM wave propagating through different paths to reach the sensor, it can result in a different time of arrival (TOA). The time difference of arrival (TDOA) is a critical parameter to determine the location of the PD source through a triangulation calculation. Compared to the acoustic method, the weakness of UHF localization is that EM waves propagate very fast in transformer oil with a velocity of approximately 2 × 10^8^ m/s [95]. Consequently, TDOA is measured in nanoseconds and if a small error exists, the estimated location of the PD source can vary dramatically [96]. Moreover, a high sampling rate measurement system up to 3 GHz is required in order to digitize the signal adequately [37,45]. It is noted that a feasible arrangement for a three-dimensional positioning system is to use four UHF sensors mounted around the tank but not all on the same plane [60].

In addition to the time-difference (TDOA) localization, other algorithms have been proposed in recent literature. The time difference is determined from the measured signals in the time domain; therefore, a high sampling rate measurement system is required. To reduce the hardware cost and amount of data to be digitized and processed, the received signal strength [45,103], direction of arrival (DOA) measurement [45,104,105] as well as the combined acoustic/UHF method [37,106] are proposed. These approaches give potential solutions in reducing the data volume and the number of sensors required to satisfy the operating requirements. Using a moving sensor can also reduce the number of sensors, which is proposed by Robles et al. [107]. However, this kind of solution may not be feasible if PDs occur at a low rate. Li et al. [45] locate the equipment having PDs in a substation with a received signal strength indicator (RSSI) based on compressed sensing [45]. This method assumes that the source position can be estimated by analyzing the amplitude of the received signal, i.e., the closest sensor to the source is able to capture signals with a higher amplitude while more distant sensors receive weaker signals [38]. Each testing point in the substation has a unique fingerprint according to the RSSI, and a matrix consisting of these fingerprints is developed as a map. When a discharge occurs within some faulty equipment, PD signals can be received by the sensors, and the corresponding position can be determined using the RSSI technique. The researchers then turned their focus to power transformers using the same RSSI technique [20]. However, this method is highly dependent upon the layout of the sensors and internal barriers along the signal propagation path. Such obstacles can significantly affect the attenuation and distortion of the received signal. This results in significant errors in the localization. Nafar et al. [108] noted that the frequency component of the neutral point current is dependent on the PD location, which provides the possibility of simplifying the PD measurement system.

## 6. Challenges and Future Development

Recently, some researchers have been developing a hybrid detection system using multiple sensing techniques in order to take advantages of various methods [109,110]. The UHF signal is an effective trigger for indicating a PD occurrence. By combining these detection methods systematically in further study, the performance of the monitoring system can be improved. In addition, due to the nature of the PD activities in the field, the occurrence of PD events commonly scatters in the equipment, so it is more likely that a discharge could occur simultaneously at different locations. The multiple sources localization and recognition techniques have been investigated recently and requires further development with regards to their accuracy and efficiency along with their PD identification capability.

## 7. Conclusions

This paper provides a detailed review of UHF sensors utilized for partial discharge detection in high voltage power system equipment. The key advantages of the UHF method in PD detection are shown by comparison with existing detection approaches in terms of their application. The fundamentals, essential for antenna design, are explained which can assist researchers in sensor design and performance evaluation for both simulation and experimental work. Moreover, comparative analysis of commercially available UHF sensors and those proposed in recent research are also presented to assist with sensor selection. The effects of transformer’s physical structure on the UHF detection are discussed as well as PD localization using a sensor array. The current challenges to widespread adoption of the UHF method are mentioned which suggest potential areas of research which could lead to improvements of the UHF method.

## Figures and Tables

**Table 1:** Partial discharge (PD) detection methods.

Method	Detection Phenomenon	Applied Sensor	PD Localization	**Online Monitoring**
IEC 60270 method	Current impulse below 1 MHz	Coupling capacitor	Yes	No
Dissolved gas analysis	Chemical reactions	Gas Chronographs	No	Yes
Acoustic method	Pressure waves	Piezoelectric sensors	Yes	Yes
High frequency (HF) method	Magnetic field	High frequency current transformer (HFCT)	Yes	Yes
Transient Earth Voltage (TEV) method	Transient earth voltage	TEV sensor	Yes	Yes
Radio frequency (RF) method	Electromagnetic wave	VHF/UHF antenna ^1^	Yes	Yes
Optical method		Optical sensor	No	Yes

^1^ VHF refers to the range of radio frequency electromagnetic waves from 30 MHz to 300 MHz.

**Table 2:** Comparison of commercial UHF sensors for PD detection.

Sensor	Frequency Range	Applied Installation	Features	Company	Ref.
IA-MM-TDP	N/A	Medium Voltage Switchgear	Wireless sensor with noise cancellation	IntelliSAW	[29]
DA100 Directional Antenna	250 MHz–1 GHz	Substation Survey	Handheld or mounted on a tripod	Doble	[30]
Telescopic Antenna	250 MHz–1.9 GHz	Substation Survey	Handheld		
Whip Antenna	250 MHz–1.9 GHz	Substation Survey	Handheld		
UCS 1	100 MHz–1 GHz	HV cable and cable termination	Not require parallel installed grounding connections	Omicron	[31]
UHT 1	200 MHz–1 GHz	Power transformer	Installed permanently on the tank surface as the internal sensor		
UVS 610	150 MHz–1 GHz	Liquid-insulated Power transformer	Matching with DN-50 and DN-80		
UHF Hatch Cover PD Sensor	200 MHz–1.2 GHz	Power transformer	External flange sensor via a dielectric window	Power Diagnostic Service	[32]
UHF CT	30 MHz–900 MHz	Cable terminations, cable joints, transformers, high voltage motors	Attached to the ground wire		
UHF Bushing PD Sensor	30 MHz–900 MHz	oil-immersed transformer and generator	Install at the bottom of the bushing		
UHF TEM PD Sensor	150 MHz–1.2 GHz	High voltage switchgear	installed inside the switchgear panel, non-contact		
UHF Drain Valve PD Sensor	200 MHz–1.2 GHz	Liquid-insulated Power transformer	Oil valve		
TFS 1	N/A	Power transformer	Valve flange	Power Diagnostix	[33]
DFS 1	N/A	Cable joints and terminations	Differential foil sensor		
TVS 2	300 MHz–1 GHz	Liquid-insulated Power transformer	Oil valve		
EFS1	N/A	GIS and Gas-insulated transmission lines	Wrapped around the unshielded flange		
WS 80/95/140	N/A	GIS	External flange sensor via a dielectric window		

**Table 3:** Comparison of UHF sensors proposed in PD detection.

Antenna Configuration	Measurement Bandwidth	Physical Size $(L){\;}^{1}$	Electrical Length $(\lambda_{f}^{*}){\;}^{2}$	Radiation Pattern	Ref.
Meander-line antenna	0.3 GHz–1 GHz	70 mm	0.07	Unidirectional	[59]
Vivaldi antenna	0.8 GHz–3 GHz	100 mm	0.27	Omnidirectional	[60,61]
Monopole antenna	0.75 GHz–1.5 GHz	100 mm	0.25	Omnidirectional	[41]
Goubau antenna	0.4 GHz–1 GHz	207 mm	0.276	Omnidirectional	[42]
Conical antenna	0.6 GHz–3 GHz	100 mm	0.20	Omnidirectional	[41,62]
Hilbert fractal antenna	0.3 GHz–1 GHz	100 mm	0.1	Unidirectional	[57]
Peano fractal antenna	0.3 GHz–1 GHz	90 mm	0.09	Unidirectional	[63,64]
Bowtie antenna	N/A	N/A		Unidirectional	[54]
U-shaped UWB antenna	0.5 GHz–1.5 GHz	215 mm	0.36	Unidirectional	[55]
Squared patch antenna	0.35 GHz–800 MHz	232 mm	0.27	Unidirectional	[65]
Log-Spiral antenna	0.7 GHz–3 GHz	150 mm	0.35	Unidirectional	[66]
Single-Arm Archimedean Spiral Antenna	1.15 GHz–2.4 GHz	200 mm	0.77	Unidirectional	[67]
Double-Arm Archimedean Spiral Antenna	0.6 MHz–1.5 GHz	130 mm	0.26	Unidirectional	[41]
Cavity-backed Archimedean Spiral Antenna	0.925 GHz–1.6 GHz	80 mm	0.25	Unidirectional	[50]
Minkowski Fractal Antenna	0.7 GHz–3 GHz	300 mm	0.70	Omnidirectional	[52]
Circular Patch Antenna	0.8 GHz–3 GHz	100 mm	0.27	Omnidirectional	[49]
3D cube antenna	1.25 GHz–3 GHz	85 mm	0.35	Unidirectional	[44]
Koch Snowflake antenna	0.3 GHz–1 GHz	280 mm	0.28	Omnidirectional	[68]

^1^ The physical size (L) is determined as the longest dimension of the antenna.

^2^ The electrical length ($\lambda_{f}^{*}$) is normalized by the physical size of the antenna, which is calculated by: (1) $$\lambda_{f}^{*}=\frac{c_{0}/f_{min}}{L},$$
where $c_{0}$ is the speed of the light; $f_{min}$ is the lowerst working frequency.

**Table 4:** Classification of the antenna based on the frequency bandwidth.

Antenna Type	Frequency Bandwidth
Narrow Bandwidth	B < 0.1
Wide Bandwidth	0.1 ≤ B ≤ 0.6
Ultra-Wide Bandwidth	B > 0.6
